# Ester Bonds for Modulation of the Mechanical Properties of Protein Hydrogels

**DOI:** 10.3390/ijms241310778

**Published:** 2023-06-28

**Authors:** Di Zhang, Luofei Li, Yizhou Fang, Quan Ma, Yi Cao, Hai Lei

**Affiliations:** Collaborative Innovation Center of Advanced Microstructures, National Laboratory of Solid State Microstructure, Department of Physics, Nanjing University, 22 Hankou Road, Nanjing 210093, China

**Keywords:** protein, ester bond, hydrogel, fatigue

## Abstract

Hydrogels are soft materials constructed of physically or chemically crosslinked polymeric net-works with abundant water. The crosslinkers, as the mechanophores that bear and respond to mechanical forces, play a critical role in determining the mechanical properties of hydrogels. Here, we use a polyprotein as the crosslinker and mechanophore to form covalent polymer hydrogels in which the toughness and fatigue fracture are controlled by the mechanical unfolding of polyproteins. The protein Parvimonas sp. (ParV) is super stable and remains folded even at forces > 2 nN; however, it can unfold under loading forces of ~100 pN at basic pH values or low calcium concentrations due to destabilization of the protein structures. Through tuning the protein unfolding by pH and calcium concentrations, the hydrogel exhibits differences in modulus, strength, and anti-fatigue fracture. We found that due to the partially unfolding of ParV, the Young’s modulus decreased at pH 9.0 or in the presence of EDTA (Ethylene Diamine Tetraacetic Acid), moreover, because partially unfolded ParV can be further completely unfolded due to the mechanically rupture of ester bond, leading to the observed hysteresis of the stretching and relaxation traces of the hydrogels, which is in line with single-molecule force spectroscopy experiments. These results display a new avenue for designing pH- or calcium-responsive hydrogels based on proteins and demonstrate the relationship between the mechanical properties of single molecules and macroscopic hydrogel networks.

## 1. Introduction

Hydrogels are three-dimensional networks of polymer chains formed through physical or chemical crosslinking and contain abundant water. Due to their unique properties, such as high water content, flexible texture, and biocompatibility, hydrogels have a wide range of applications in various fields, including soft robotics [1,2], contact lenses [3,4], drug-delivery systems [5,6], and tissue-engineering scaffolds [7,8]. These applications are mainly determined by the mechanical properties of hydrogels (e.g., modulus and anti-fatigue fracture). Thus, the rational design of hydrogels with proper mechanical properties from bottom to top is of prime importance.

As the scaffold of a hydrogel is composed of polymers and crosslinking molecules, the crosslinkers serve to physically hold the polymer chains in place, which provides the hydrogel with unique mechanical and chemical properties. Crosslinkers can be classified into two main types: physical and chemical. Physical crosslinkers rely on noncovalent interactions to hold the polymer chains together [9,10,11,12]. Chemical crosslinkers [13,14,15], on the other hand, form covalent bonds or dynamic bonds [16,17] between the polymer chains, which are much stronger and more durable. The properties of the crosslinker can have a significant impact on the properties of the resulting hydrogel [18,19]. For example, [the length of the crosslinker can affect the density of the network [20,21], with shorter crosslinkers resulting in a more tightly packed network. Similarly, the concentration of the crosslinker can affect the porosity and water content of the hydrogel [22,23]. Moreover, by incorporating mechanophores that contain specific chemical bonds or groups that can undergo a mechanical reaction when subjected to forces into the polymer network of a hydrogel, researchers can create materials that can respond to mechanical stimuli such as stretching or compression by undergoing a specific chemical or physical change [24]. These molecular events, such as bond cleavage or conformational change, provide a molecular-scale reading of the local mechanical state or transformation of material properties in response to the local mechanical environment. However, how to connect the molecular behavior and the macroscopic properties is challenging.

In recent years, there has been a growing interest in understanding the role of DNA [25,26,27,28] and proteins [29,30] in determining the mechanical properties of hydrogels. The precise base pairing of DNA allows it to become a versatile building block for rationally designing hydrogels with programmable response and function. For example, Liu et al. incorporated polymetric multiple-unit linker into a polymeric backbone to reinforce the mechanical properties of DNA supramolecular hydrogels [31]. Proteins are known to be key players in the mechanical properties of biological tissues through conformational change [32,33,34], and their incorporation as crosslinkers or mechanophores into hydrogels has been shown to significantly tune the mechanical performance [35]. This has led to the development of protein-based hydrogels that can be tailored to mimic the properties of specific tissues, making them a promising platform for various biomedical applications [36,37]. Thanks to the development of single-molecule force spectroscopy (SMFS) [38,39,40], we can precisely obtain the mechanical unfolding/folding forces of proteins and coiled coils interactions [41]. Therefore, it is a good way to study the connection between the two different-length scales, molecular scale and bulk, by incorporating proteins into hydrogels.

**Figure 1:**
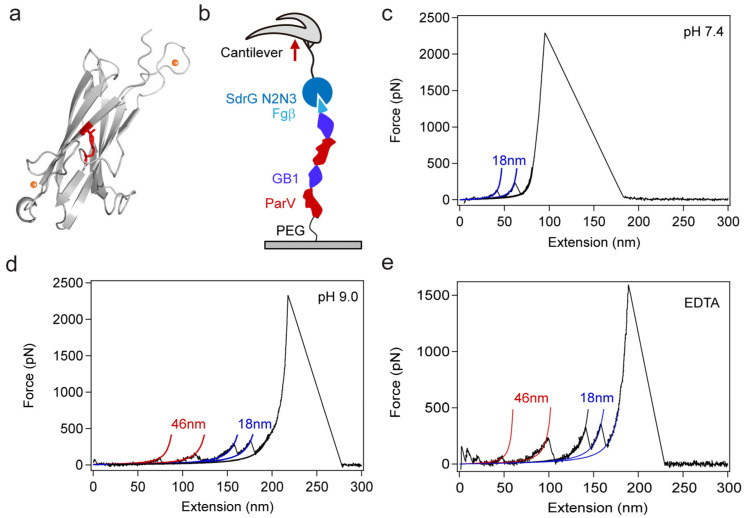
Mechanical unfolding of ParV by using AFM-based single-molecule force spectroscopy experiments. (**a**) Structure of the ParV protein, ester bond is shown in red; calcium ions are shown as orange spheres; (**b**) schematic of the AFM-based SMFS experiments. Fgβ-(GB1-ParV)_2_-cys was covalently linked to the substrate through a polymer linker via thiol-maleimide chemistry and picked up by a SdrG-cys-modified cantilever; (**c**) representative single-molecule force-extension curves with a pulling speed of 1.6 μm s^−1^ at pH 7.4 showing the unfolding of GB1 domains but no unfolding events of ParV up to the dissociation of the Fgβ-SdrG complex (>2 nN). The curves are fitted by the worm-like chain model; (**d**,**e**) representative force-extension curves at pH 9.0 and with 10 mM EDTA, respectively. Except for the two GB1 unfolding events, there are two additional peaks with Δ*L_c_* 46 nm corresponding to the unfolding of ParV.

Here, we use the ester-containing protein ParV from the gram-positive bacterium Parvimonas sp. as the model system (Figure 1a) [42]. Combining protein engineering and SMFS, we found that protein ParV is super mechanically stable and maintains folding even at a pulling force > 2 nN. However, the basic pH and low calcium concentration can destabilize the protein structure, resulting in unfolding forces of ~100 pN. These results are comparable to our previous study of another ester-containing protein, Cpe0147. Then, we used the pH- or calcium-dependent mechanical unfolding of protein ParV as a crosslinker and mechanophore to form a covalent hydrogel. The results show that the reactivity of the protein dictates the modulus and anti-fatigue fracture of the hydrogel. We anticipate that this can be used as a guideline for the rational design of hydrogels.

## 2. Results and Discussion

To investigate the mechanical stability of protein ParV, we engineered a chimeric protein, Fgβ-(GB1-ParV)_2_-cys (Figures S1 and S2), for SMFS experiments (Figure 1b), following our previous experimental protocol [43]. In brief, the polyprotein is covalently linked to the substrate through the thiol group of C-terminal Cys and then picked up by the cantilever modified with protein SdrG via strong noncovalent interactions between Fgβ and SdrG. The unfolding of the GB1 domain was characterized by a contour length increment of ~18 nm, and an unfolding force of ~200 pN at a pulling speed of 1.6 μm/s acted as a fingerprint in SMFS [38,44]. The protein ParV contains an ester bond and two calcium ions, similar to the protein Cpe0147 we previously studied, whose mechanical stability is modulated by pH and calcium concentration [43]. Therefore, we performed SMFS experiments under different physiological conditions.

First, we stretched the polyprotein in Tris buffer at pH 7.4, and the typical sawtooth-like force-extension curves are shown in Figure 1c, in which each individual sawtooth peak corresponds to the force-induced unfolding of individual domains in the polyprotein chain. As expected, there are only three peaks, two of which showed the same contour length increments (Δ*L_c_*) of 18 nm, which can be attributed to the unfolding of the two GB1 domains. The last peak of more than 2 nN arises from the unbinding of the Fgβ/SdrG interaction. There was no other peak observed. The initial contour length is 32.6 ± 12.3 nm (Figure S3), corresponding well to the theoretically estimated value that includes the contour length of the PEG(Polyethylene glycol) linker (~30 nm), the two folded GB1 (~5 nm), and two folded ParV (~5 nm). These results indicated that ParV did not fully unfold even under a force > 2 nN. Next, we performed pulling experiments in Tris buffer at pH 9.0. Different from that at pH 7.4, the force-extension curves displayed five sawtooth peaks, as shown in Figure 1d. Two peaks with contour length increments of 46 nm occur first, followed by two peaks with Δ*L_c_* ~ 18 nm for GB1. The additional peak of Δ*L_c_* at 46 nm is consistent with the theoretical calculation (134 aa × 0.36 nm/aa–1.32 nm, the number of caged residues by ester bond is 134 and the distance between the residues forming ester bond is 1.32 nm), which is attributed to the unfolding of ParV. The mechanical stability of ParV was reduced, and the unfolding force of ParV was reduced at pH 9.0. In addition, we added 10 mM EDTA to Tris buffer at pH 7.4 to conduct SMFS experiments. The force-extension curves also displayed five sawtooth peaks, as shown in Figure 1e, the same as that of pH 9.0. The calcium chelation of EDTA destabilized the structure of ParV, resulting in unfolding forces of ~100 pN. The histograms of the contour length increments and the rupture forces at pH 9.0 and pH 7.4 with EDTA are shown in Figure S4. The frequency of the ester bond rupture is ~33.5% (91/268) and 15.3% (37/242) at pH 9.0 and pH 7.4 with EDTA, respectively. These results demonstrate that pH and calcium can modulate the mechanical stability of the ester-containing protein ParV, which is in accordance with the underlying mechanism of unfolding of Cpe0147. Our results confirmed that the mechanical stability of ester bond-containing proteins of this type is a conserved feature in cell-surface proteins of bacteria. After all, the mechanically adjustable protein of ParV should be a good candidate to study the relationship between molecular scale and macroscopic scale in hydrogel materials.

**Figure 2:**
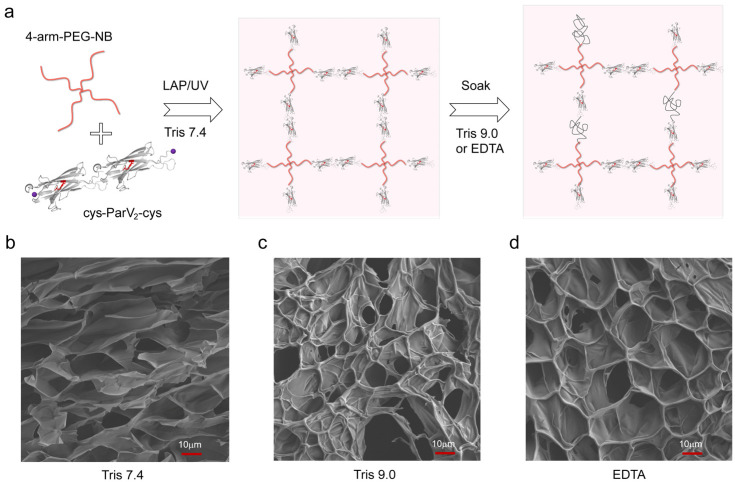
Responsive covalent hydrogel formed by polyprotein cys-ParV_2_-cys with 4-arm-PEG-NB. (**a**) Schematic illustration of the preparation of the covalent hydrogel. The hydrogel is prepared by the photoclick reaction of polyprotein and PEG using UV illumination and LAP as initiators in Tris buffer at pH 7.4. After sufficient swelling, the hydrogels were soaked in different conditions: Tris buffer at pH 7.4, Tris buffer at pH 9.0, and Tris buffer at pH 7.4 with 10 mM EDTA; (**b**–**d**) SEM images of the lyophilized hydrogels.

After characterizing the mechanical properties of ParV, we employed it as a crosslinker and mechanophore to form a hydrogel. To directly probe the role of protein in determining the mechanical properties of macroscopic hydrogels, we incorporated the dimer of ParV into covalent polymer networks, as shown in Figure 2a. The hydrogels were prepared by the polymerization of cys-ParV_2_-cys and four-armed norbornene-terminated polyethylene glycol (4-arm-PEG-NB) through thiol-norbornene photoclick chemistry in Tris buffer at pH 7.4 for 30 min using lithium phenyl-2,4,6-trimethylbenzoylphosphinate (LAP) as the photoinitiator [45,46]. Then, the hydrogels were dialyzed against Tris buffer at pH 7.4 for 24 h to completely remove all undesired byproducts or unreacted reactants. In the following, the hydrogels were soaked in three different kinds of solutions, Tris buffer at pH 7.4, Tris buffer at 9.0, and Tris buffer at pH 7.4 with 10 mM EDTA. By doing so, these hydrogels should exhibit different mechanical characteristics due to the pH/EDTA-modulated unfolding of proteins.

The scanning electron microscopy (SEM) images revealed that the PEG-protein conjugates formed highly porous networks at the nanoscale in all three conditions, as shown in Figure 2b–d. Large pores were visible on the surface of the hydrogel at pH9.0 or with EDTA. The change of the pore size might be the result of conformational change of the polyprotein crosslinkers. However, how such nanometer-scale molecular change affect the micrometer-scale hydrogel structural change remains unknown. When subjected to high pH or EDTA, the structure of protein ParV became unstable and unfolded under the swelling forces of hydrogels, resulting in an increase in crosslinking length, which led to pore enlargement. The previous results indicated that the fragments of fully unfolding ester bond-containing protein in the presence of EDTA or at pH 9.0 are 20% or 38%, respectively [43]. The swelling ratio test verified this variation. The swelling ratios of these hydrogels are different: 101% ± 5%, 203% ± 4%, and 131% ± 5%, respectively (Figure S5a). These results suggested that there must be differences in the mechanical properties that are related to the behavior of protein unfolding.

**Figure 3:**
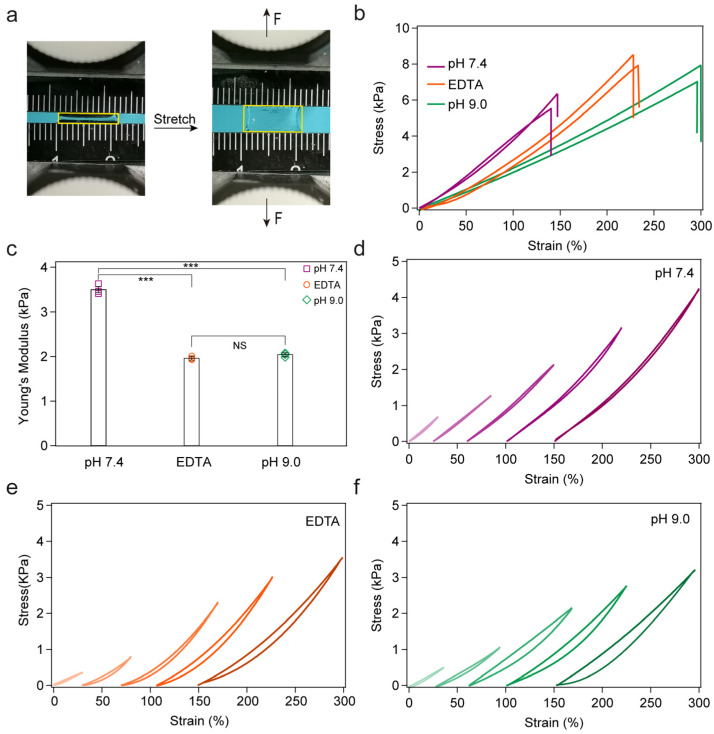
The bulk mechanical properties of the hydrogels. (**a**) Tensile test experiments. The yellow rectangles indicate the hydrogel; (**b**) stress–strain curves for hydrogels under different conditions; (**c**) Young’s modulus of hydrogels in all three conditions; the numbers of hydrogels in all conditions are 3. (**d**–**f**) Representative stretching-relaxation curves at different strains for hydrogels. The curves are horizontally offset for clarity. *** indicates *p* < 0.05.

Next, we evaluated the effect of embedded polyprotein on the mechanical properties of hydrogels using tensile test experiments (Figure 3a). All mechanical properties were tested in air at room temperature at a constant tensile rate of 5 mm/min unless otherwise noted. The stress–strain curves of these three kinds of hydrogels are shown in Figure 3b. The hydrogel at pH 7.4 can only be extended ~1.5 times its original length, while the other two hydrogels displayed lengths of over two or even three times over initial length. The tangent Young’s modulus at 5% strain also exhibits a difference, as shown in Figure 3c. The hydrogels at pH 9.0 or with EDTA became soft, and the modulus declined by half compared to that at pH 7.4. Noted that, unfolding proteins can lead to dramatic decrease in the Young’s modulus of protein hydrogels in our previous study [47]. However, this is not the case of ParV hydrogels. The ester bonds lock the protein ParV to partially unfolded conformation and prevent complete unfolding of ParV. This is also different from those reported by Li and coworkers [48] showing that the completely unfolded proteins can aggregate to increase the stiffness of the hydrogels. The stretchability and Young’s modulus are both associated with the crosslinking density of the hydrogels, in which the heavily crosslinked hydrogels are typically stiff but poorly stretchable. In our system, the hydrogels maintained the same components; however, the crosslinking was tuned. The crosslinker polyprotein bears the loading force as a mechanophore when stretching the hydrogel. In Tris at pH 7.4, the protein ParV is super stable, which is equal to covalent bonding, and is hard to unfold (Figure 1c). In contrast, the mechanical stability of protein ParV is decreased in Tris at pH 9.0 or with EDTA, which results in the unfolding and release of chain length (Figure 1d,e) when subjected to stretch in hydrogels. Thus, the crosslinking density decreased due to tuning of the basic pH or EDTA. For comparison, we used a pH-independent polyprotein cys-GB1_2_-cys to replace cys-ParV_2_-cys to form hydrogels. As shown in the Figure S5b, there were no significant differences in the swelling ratios. The tensile test experiments indicated that the mechanical properties of cys-GB1_2_-cys hydrogels in different environments were almost identical (Figure S6).

To further investigate the role of mechanical unfolding of polyproteins in hydrogels, we performed the load/unload cyclic test on all three hydrogels to different strains. Because there is no unfolding of ParV domains in Tris at pH 7.4, the stretching–relaxation cycles showed almost no hysteresis (Figure 3d). In contrast, the hydrogels in Tris at pH 9.0 or with EDTA showed clear hysteresis, which increased with increasing strain (Figure 3e,f). The hysteresis is mainly due to the unfolding of protein ParV. This further confirmed the regulation of protein unfolding at the molecular level in determining the mechanical properties of macroscopic hydrogels.

**Figure 4:**
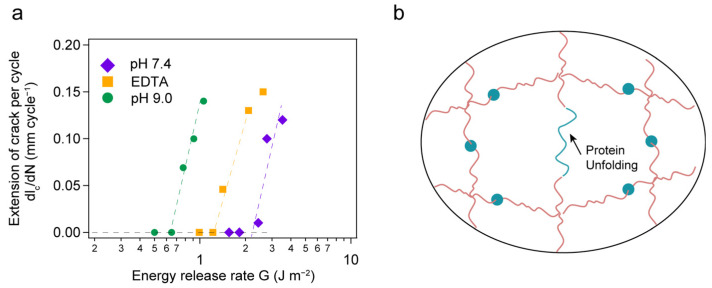
(**a**) Extension of crack per cycle as a function of the energy release rate; (**b**) schematic of the protein hydrogel network sample in pH 9.0 or with EDTA, having a topological defect induced by the unfolding of protein.

In addition, we measured the anti-fatigue fracture properties of the hydrogels under different conditions in cyclic load/unload experiments following previously published test procedures [19]. Figure 4a shows the extension of cracks per cycle as a function of the energy release rate. The fatigue thresholds of the hydrogels under different conditions are 2.16, 0.64 and 1.12 J m^−2^, respectively. The difference in fatigue thresholds is merely attributed to the molecular mechanophore present in each linker. Typically, the fracture threshold can be described by using the well-known Lake–Thomas theory [49]:

$$\Gamma =\sigma W=\frac{1}{2}\nu_{0}R_{0}nU,$$

where $R_{0}$ is the average end-to-end distance of an elastically active network strand in its undeformed state, $\nu_{0}$ is the number density of such elastically active subchains, *n* is the average number of repeat units along the bridging strand, and *U* is the energy that is stored in each repeat unit when the bridging strand breaks. The prefactor of 1/2 comes from the projection of the end-to-end vectors of subchains onto the normal of the crack plane. Further studies indicated that the original Lake–Thomas theory is not completed and expanded by considering complexity [50,51,52]. It underestimates the threshold due to the lack of effects such as the energy stored in multiple layers of polymer chains adjacent to the crack or viscoelastic dissipation.

However, the existing theory cannot depict the fracture threshold in our system. For the hydrogel at pH 7.4, due to the super mechanical stability of protein ParV, it would not unfold until the chain breaks, resulting in crack propagation. The polyprotein can be regarded as a covalent bond in the hydrogel network. For the hydrogels at pH 9.0 or with EDTA, the protein ParV was destabilized because of ester bond hydrolysis and could be unfolded when stretching. According to the Lake–Thomas theory, the number density $\nu_{0}$ decreased while the end-to-end distance $R_{0}$ increased as well as the repeat number *n*, tuned by the high pH and EDTA. The intuition told us that the fracture threshold could be increased greatly owing to the energy release of protein unfolding. In fact, Stephen L. Craig et al. designed a toughening hydrogel through force-triggered chemical reactions by using a mechanophore in which the process is similar to protein unfolding upon stretching [24]. The hydrogels exhibited an extreme fatigue threshold compared to the control. In contrast, in the hydrogel with unfolded protein at pH 9.0 or with EDTA in our system, the fracture threshold decreased. This might also be related to the mechanical unfolding kinetics of the protein. Protein mechanical folding/unfolding is a Markovian process that depends on the loading rates. Thus, the destabilized protein in pH 9.0 or with EDTA will unfold during swelling of the hydrogel, which results in networks with topological “defects” (shown in Figure 4b). This inactive “defect” led to an increase in the swelling ratio and decreased the overall intrinsic fracture energy of the hydrogel network [50,53].

**Figure 5:**
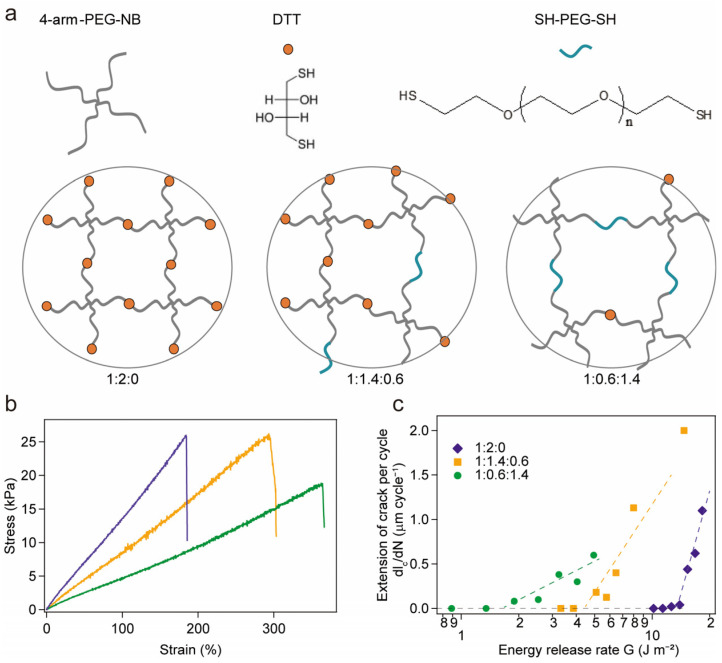
(**a**) Schematic illustrations of the synthesis and structures of hydrogels with different ratios in 4-arm-PEG-NB, DTT and SH-PEG-SH through the thiol-norbornene photoclick chemistry; (**b**) stress–strain curves of the hydrogels in different ratios; and (**c**) extension of crack per cycle as a function of the energy release rate.

To study the defect effect in the hydrogel, we prepared covalent hydrogels by using 4-arm-PEG-NB, dithiothreitol (DTT) and SH-PEG-SH through a thiol-norbornene photoclick chemistry with various ratios, as shown in Figure 5a. The monomer molar ratios of 4-arm-PEG-NB, DTT and SH-PEG-SH are 1:2:0, 1:1.4:0.6 and 1:0.6:1.4, respectively. DTT can be regarded as the folded protein and SH-PEG-SH as the unfolded protein in our system. In this way, the crosslinker SH-PEG-SH can be regarded as a defect in the hydrogel network. The stress–strain curves of these three hydrogels are shown in Figure 5b, and the fatigue fracture energies are shown in Figure 5c. As expected, the fatigue of the hydrogel with a ratio of 1:2:0 is much higher than that of the others, and the fatigue decreased with the addition of SH-PEG-SH. This result is similar to that of the protein-based hydrogels in our system and confirms the defect effect in determining the fatigue fracture energy.

## 3. Materials and Methods

### 3.1. Materials

(3-Aminopropyl)triethoxysilane (APTES) was purchased from Sigma-Aldrich. (Merck KGaA, Darmstadt, Germany) Maleimide-PEG-NHS(MAL-PEG-NHS, MW 5 kDa) was purchased from Nanocs, Inc. (New York, NY, USA). 4-arm-PEG-NB (MW 20 kDa) was purchased from Shanghai ToYongBio Tech. Inc. (Shanghai, China) Lithium phenyl-2,4,6-trimethylbenzoylphosphinate (LAP) was purchased from Tokyo Chemical Ind. (Tokyo, Japan) All the other chemicals were purchased from Sinopharm Chemical Reagent Co., Ltd. (Beijing, China).

### 3.2. Protein Expression and Purification

The genes encoding the SdrG-cys and Fgβ-(GB1-ParV)_2_-cys proteins were cloned into the pET22b vector and the genes encoding the cys-GB1_2_-cys and cys-ParV_2_-cys proteins were cloned into the pQE80L vector. All the proteins were expressed in *E. coli* BL21 and purified with Co^2+^ affinity chromatography. The purified cys-ParV_2_-cys and cys-GB1_2_-cys protein were dialyzed against pure water and then freeze-dried for preservation until use. The purified Fgβ-(GB1-ParV)_2_-cys and SdrG-cys proteins were stored in Tris-HCl buffer for SMFS experiments.

### 3.3. Single-Molecule Force Spectroscopy (SMFS) Experiments

SMFS experiments were performed by using a commercial atomic force microscopy (NanoWizard-IV, JPK, Berlin, Germany). The MLCT-D cantilever was used to conduct experiments. The experiments were carried out at a speed of 1.6 μm/s at room temperature in different buffers, including Tris-NaCl-pH 7.4 buffer (50 mM Tris, 10 mM NaCl, pH 7.4), Tris-NaCl-pH 9.0 buffer (50 mM Tris, 10 mM NaCl, pH 9.0), and Tris-EDTA-pH 7.4 buffer (50 mM Tris, 10 mM EDTA, pH 7.4). To efficiently obtain force-extension curves during pulling experiments, the glass substrates and cantilevers were chemically modified.

Glass substrate modification: 1 × 1 cm^2^ glass substrates were placed in chromic acid solution overnight to hydroxylate the surface. Then, the glass substrates were washed successively with water and ethanol, and blown dry with N_2_. Next, the cleaned glass substrates were immersed in a toluene solution containing 1% APTES for 1 h. After washing the substrates with toluene and ethanol, the substrates were placed in an oven at 80 °C for 0.5 h. The substrates were cooled to room temperature and dipped in dimethyl sulfoxide (DMSO) solution with 1 mg/mL MAL-PEG-NHS for 1 h. Finally, the maleimide-modified glass substrates were washed with DMSO and ethanol and stored in a sealed pot with argon after blowing dry with N_2_ until use in SMFS experiments. For SMFS experiments, typically 100 μL of 2 mg/mL Fgβ-(GB1-ParV)_2_-cys solution was pipetted onto the glass surface to incubate for 1 h and then the substrates were washed with Tris-HCl buffer. Substrates modified with proteins were prepared.

Cantilever modification: The cantilever was exposed to chromic acid solution at 80 °C for 0.5 h and washed successively with water and ethanol. Then, the cantilever was immersed in the APTES solution (1% APTES in toluene) for 1 h. After that, the cantilevers were washed successively with toluene and ethanol and incubated at 80 °C for 0.5 h. Next, the cantilevers were transferred into DMSO solution with 1 mg/mL MAL-PEG-NHS for 1 h. After washing with DMSO and ethanol, the cantilevers were dipped in 4 mg/mL SdrG-cys solution for 1 h and then washed with Tris-HCl buffer. The cantilevers were prepared for use.

### 3.4. Preparation of the Hydrogels

To prepare the hydrogels, 4-arm-PEG-NB (30 mg/mL) and polyprotein cys-ParV_2_-cys or cys-GB1_2_-cys were mixed with a molar ratio of 1:2 in the presence of 1 mg/mL LAP. After centrifugation, the mixture was transferred to a custom-made transparent glass mold with a thickness of 1 mm. Photoclick chemistry was performed under UV (365 nm, 7.526 mW/cm^2^) illumination for 0.5 h at room temperature, which is unlikely to introduce the conformational change of proteins according to literature [54]. Subsequently, the formed hydrogels were removed from the mold and soaked in Tris buffer at pH 7.4 and 4 °C for 24 h to reach the equilibrium–swollen state. Then, the hydrogels were immersed in different solutions, Tris buffer at pH 7.4, Tris buffer at pH 9.0, and Tris buffer at pH 7.4 with 10 mM EDTA, for 24 h.

### 3.5. Tensile Test

The tensile stress–strain measurements were performed using a tensile tester (Instron 5944 with a 10 N sensor) in air at room temperature. For the tensile test, the samples had a rectangle shape, typically 8.0 mm in length and 10.0 mm in width. In the tensile tests, the velocity of stretching is 5 mm min^−1^. The nominal strain was measured through the distance between clamps (typically 2.0 mm) and the nominal stress was calculated as the measured load divided by the original cross-sectional area vertical to the load. The fatigue behaviors of the hydrogels were measured upon cyclic load/unload tests, as shown in Figure S7. The energy release rate G of the notched hydrogel can be calculated by G = HW(λ), where H is the distance between the clamps when the hydrogel is undeformed, and W(λ) is obtained by integrating the area below the stress–stretch curve of the unnotched sample. The fatigue threshold of the hydrogel can be described as Γ = HW(λc); λc is defined as the stretch just when the crack begins to propagate noticeably. During the load/unload test with different stretch λ, we controlled the frequency as 1 Hz and the total cycle number is 5000 for all tests. Then, we recorded the pictures of the hydrogel every 100 cycles to calculate the extension of crack per cycle under specific λ. Through the approach method, we obtained the critical stretch λc and the fatigue threshold is calculated.

## 4. Conclusions

In summary, we employed an ester bond-containing protein as a crosslinker and mechanophore in a covalent hydrogel network to reveal the relationship between mechanical properties on the molecular scale and macroscopic scale. The protein ParV remains folded at pH 7.4 even at a loading force equal to the covalent bond (>2 nN); however, it unfolds at a much lower force (~100 pN) when subjected to high pH or with EDTA. By tuning the mechanical protein unfolding through high pH or the addition of EDTA, the mechanical properties of the hydrogel were significantly altered, such as the swelling ratio, Young’s modulus, hysteresis, and fracture energy. Hydrogels with tunable mechanical properties can be promising materials for many applications, including healthcare, soft robots, and environmental science. Our results suggest the important role of molecules in determining the mechanical properties of bulk hydrogels, especially for the incorporation of mechanophores. Therefore, developing an effective theory to predict the mechanical properties is of critical importance. However, accurately evaluating the fracture energy is challenging. Experimental and theoretical efforts are currently under way to address this important challenge.

## Data Availability

Not applicable.

## Supplementary Materials

The following supporting information can be downloaded at: https://www.mdpi.com/article/10.3390/ijms241310778/s1.
