# Supplementary material for: Ester Bonds for Modulation of the Mechanical Properties of Protein Hydrogels
Source: Int J Mol Sci. 2023 Jun 28;24(13):10778. doi: 10.3390/ijms241310778 (PMC10341797; doi:10.3390/ijms241310778)
Supplement: Supplementary file 1 [file ijms-24-10778-s001.zip › ijms-2441407-supplementary.pdf]

Supplementary Information

Ester Bonds for Modulation of the Mechanical Properties of Protein Hydrogels

Di Zhang, Luofei Li, Yizhou Fang, Quan Ma, Yi Cao * and Hai Lei *

Collaborative Innovation Center of Advanced Microstructures, National Laboratory of Solid State
Microstructure, Department of Physics, Nanjing University, 22 Hankou Road, Nanjing 210093, China; njuzdi@163.com (D.Z.); luffy19980620@gmail.com (L.L.); chex408fyz@163.com (Y.F.);
maquan@smail.nju.edu.cn (Q.M.)
* Correspondence: caoyi@nju.edu.cn (Y.C.); leihai@nju.edu.cn (H.L.)

Fgβ-(GB1-ParV)$_2$-cys:

MKLGSNEEGFFSARGHRPLDGSGSGSGSAGTGSGRSMDTYKLILNGKTLK
GETTTEAVDAATAEKVFKQYANDNGVDGEWTYDDATKTFTVTERSGEIKT
TVMAGGKTSTENEVATLKAKDIEGGVEVSDKITYKGLYPNEKYDVIGKIY
EVKDGELVNPGHPVSVVNSGDDLKADTTGKGEWTLNFGKLNLEAGKSYVV
FETVVSMENVIDTDGDGKPDKKQELTHEDPNDKSQTFRIRSMDTYKLILN
GKTLKGETTTEAVDAATAEKVFKQYANDNGVDGEWTYDDATKTFTVTERS
GEIKTTVMAGGKTSTENEVATLKAKDIEGGVEVSDKITYKGLYPNEKYDV
IGKIYEVKDGELVNPGHPVSVVNSGDDLKADTTGKGEWTLNFGKLNLEAG
KSYVVFETVVSMENVIDTDGDGKPDKKQELTHEDPNDKSQTFRIRSCGTE
FAAALEHHHHHH

SdrG-cys:

MKLGSEQGSNVNHLIKVTDQSITEGYDDSDGIIKAHDAENLIYDVTFEVDD
KVKSGDTMTVNIDKNTVPSDLTDSFAIPKIKDNSGEIIATGTYDNTNKQIT
YTFTDYVDKYENIKAHLKLTSYIDKSKVPNNNTKLDVEYKTALSSVNKTIT
VEYQKPNENRTANLQSMFTNIDTKNHTVEQTIYINPLRYSAKETNVNISGN
GDEGSTIIDDSTIIKVYKVGDNQNLPDSNRIYDYSEYEDVTNDDYAQLGNN
NDVNINFGNIDSPYIIKVISKYDPNKDDYTTIQQTVTMQTTINEYTGEFRT
ASYDNTIAFSTSSGQGQGDLPPEKTRSCGTEFAAALEHHHHHH

cys-ParV$_2$-cys:

MRGSHHHHHHCGSGEIKTTVMAGGKTSTENEVATLKAKDIEGGVEVSDKIT
YKGLYPNEKYDVIGKIYEVKDGELVNPGHPVSVVNSGDDLKADTTGKGEWT
LNFGKLNLEAGKSYVVFETVVSMENVIDTDGDGKPDKKQELTHEDPNDKSQ
TFRIRSGEIKTTVMAGGKTSTENEVATLKAKDIEGGVEVSDKITYKGLYPN
EKYDVIGKIYEVKDGELVNPGHPVSVVNSGDDLKADTTGKGEWTLNFGKLN
LEAGKSYVVFETVVSMENVIDTDGDGKPDKKQELTHEDPNDKSQTFRIRSG
TC

cys-GB1$_2$-cys:

MRGSHHHHHHCGSMDTYKLILNGKTLKGETTTEAVDAATAEKVFKQYANDN
GVDGEWTYDDATKTFTVTEGPQGIWGQMDTYKLILNGKTLKGETTTEAVDA
ATAEKVFKQYANDNGVDGEWTYDDATKTFTVTERSGTC

Figure S1. Sequences for proteins Fgβ-(GB1-ParV)$_2$-cys, SdrG-cys, cys-ParV$_2$-cys and cys-GB1$_2$-cys. The sequence of ParV is shown in green and the ester bond within ParV is formed between T and Q residues.

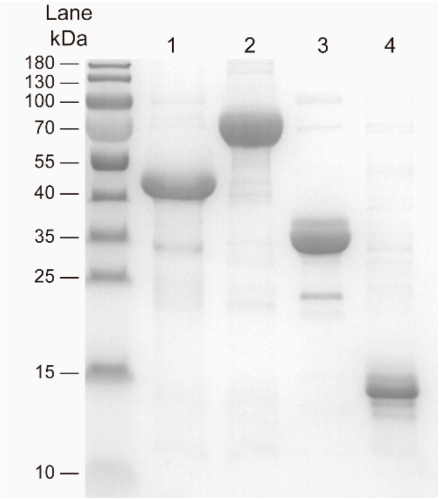

Figure S2. Proteins characterization by SDS-PAGE. Lanes 1-4 are SdrG-cys, Fgβ-(GB1-ParV)$_2$-cys, cys-ParV$_2$-cys, cys-GB1$_2$-cys, respectively.

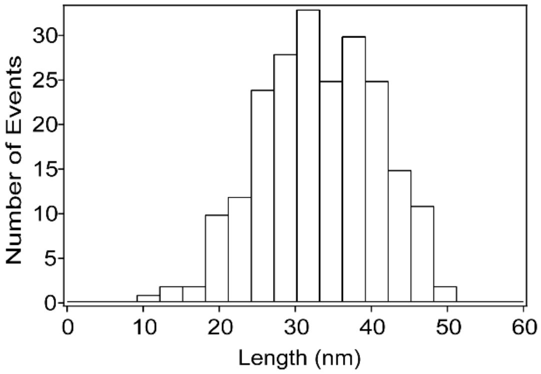

Figure S3. The histogram of the contour length before GB1 unfolding in Tris buffer at pH 7.4.

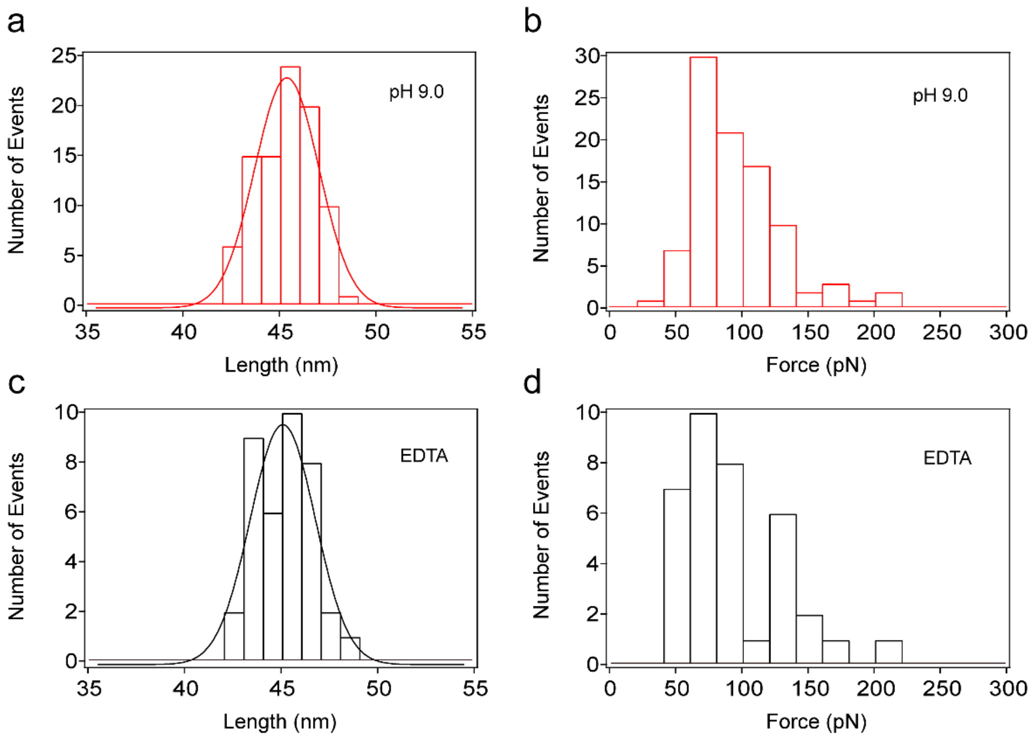

Figure S4. (**a**) Histogram of contour length increments (45.8 ± 2.32 nm) for the ester bond rupture events peaks at pH 9.0; (**b**) rupture force histogram of ester bond at pH 9.0; (**c**) histogram of contour length increments (45.4 ± 2.38 nm) for the ester bond rupture events peaks in Tris buffer with EDTA. (**d**) rupture force histogram of ester bond in Tris buffer with EDTA.

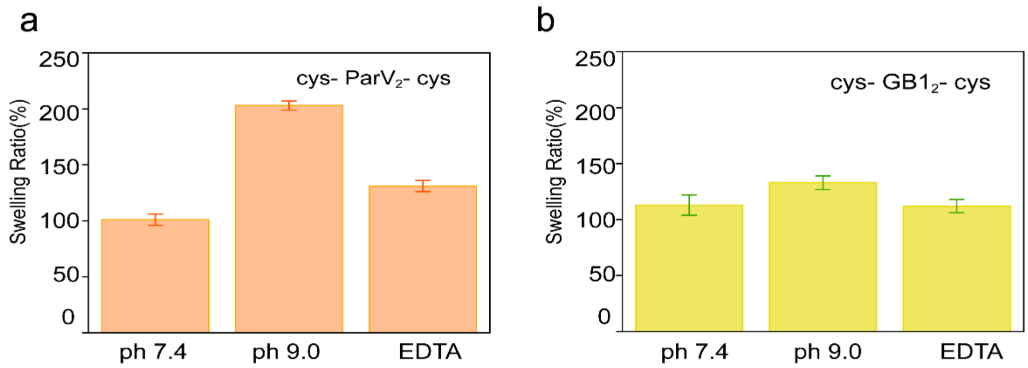

Figure S5. (a) Swelling measurement of cys-ParV2-cys hydrogels under different conditions.The swelling ratios of the hydrogels at pH 7.4, at pH 9.0 and with 10 mM EDTA are as follows: 101% ± 5%, 203% ± 4% and 131% ± 5%, respectively; (**b**) Swelling measurement of cys-GB12-cys hydrogels under different conditions. The swelling ratios of the hydrogels at pH 7.4, at pH 9.0 and with 10 mM EDTA are as follows: 113% ± 9%, 133% ± 6% and 112% ± 6%, respectively. The hydrogels were taken out and weighed immediately after being removed from the mold, and the weight was recorded as W_0. After swelling sufficiently and being immersed in different solutions, the hydrogels were removed and weighed again, with the weight recorded as W_t. The swelling ratio was calculated using following formula: Swelling ratio (%) = $[(W_t - W_0)/W_0] \times 100\%$.

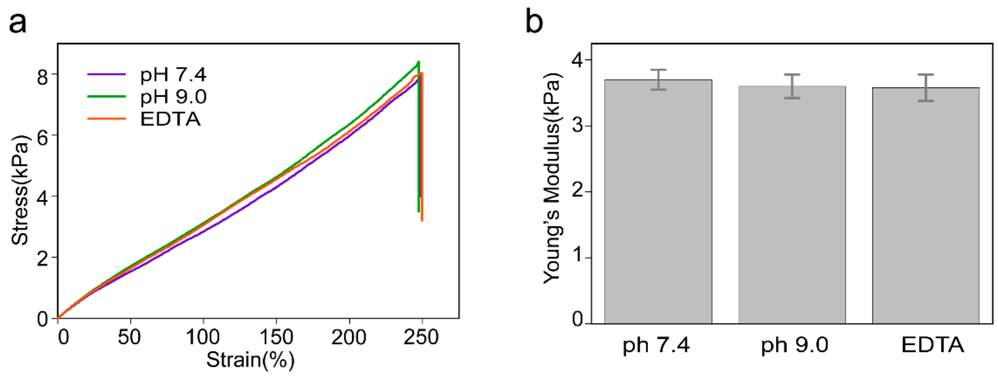

Figure S6. Tensile test experiments of cys-GB1$_2$-cys hydrogels. (**a**) Stress–strain curves for cys-GB1$_2$-cys hydrogels under different conditions; (**b**) Young's modulus of cys-GB1$_2$-cys hydrogels in three conditions.

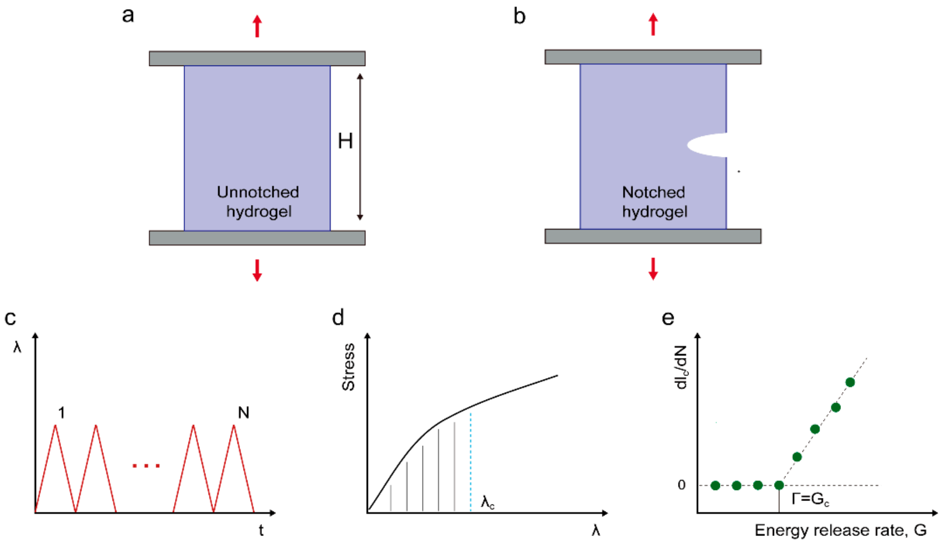

Figure S7. (**a**) Schematic of stretching unnotched hydrogel; (**b**) schematic of stretching notched hydrogel; (**c**) cyclic load/unload test; (**d**) stretch-strength curves; (**e**) illustration of measuring crack extension per cycle dl_c/dN versus energy release rate G curves.
